# Inclusive Education for Health: Analysis of the Mandatory Nature of Vaccination and Its Regulation by Administrations, and Implications and Considerations for Vaccination against COVID

**DOI:** 10.3390/vaccines10010073

**Published:** 2022-01-03

**Authors:** Eduardo García-Toledano, Emilio López-Parra, Antonio Cebrián-Martínez, Ascensión Palomares-Ruiz

**Affiliations:** Department of Pedagogy, Universidad de Castilla-La Mancha, 02071 Albacete, Spain; toledanoeg@gmail.com (E.G.-T.); emilio.lopezparra@uclm.es (E.L.-P.); antonio.cebrian@uclm.es (A.C.-M.)

**Keywords:** vaccination, COVID-19, obligation, health education, vaccination cards, equity, regulation, prevention, woman

## Abstract

In the process of vaccination against COVID-19, the problem of parents who do not want to vaccinate their school-age children has been evident. A conflict arises between two fundamental rights: the right to ideological freedom, privacy, and physical integrity of parents and minors who do not opt for vaccination; and the right to health of the rest of the children who attend the same school, provoking a social debate on the need to introduce regulatory changes that favor the mandatory imposition of vaccination in certain cases. This research offers an observational study through a cross-sectional design of a quantitative nature, in which one thousand people belonging to the education, health, and economy sectors from seventy-six countries of five continents have participated. The instrument used was a previously validated questionnaire: VACUNASEDUCA. It was considered essential to know the awareness of vaccination processes of professionals from essential social sectors. Therefore, the objectives were: to reflect on the measures of mandatory vaccination, to know the importance of teachers being able to demand a regulated vaccination card from students, to study the need for regulation by administrations of compliance with vaccines, and to analyze the importance of health education. It has been shown that women and those under thirty years of age are the least in favor of compulsory vaccination, with the health sector being the most defending of their demand, and with Europe with the lowest means. It is concluded that mandatory vaccination could be an acceptable tactical option to prevent high-risk situations.

## 1. Introduction

Childhood vaccines save more than 3 million lives a year by generating the antibodies needed to fight very serious diseases such as measles, polio, and pneumonia [1,2]. The health emergency caused by the COVID-19 pandemic, declared by the World Health Organization (WHO) in March 2020 [3], as well as its expansion throughout the world, has caused significant social, economic, and educational changes, demonstrating the importance of and need for vaccination as an effective tool to prevent the spread of diseases [4,5,6].

The principles of the European Pillar of Social Rights proclaimed at the 2017 Gothenburg Summit [7] recognize, as the first principle, inclusive and quality “Education, training and lifelong learning” in order to maintain and acquire skills that enable one to participate fully in society and successfully manage labor market transitions [8]. Similarly, principle sixteen reflects that all people have the right to timely access to affordable, preventive, and curative health care of good quality. It should be stressed that a comprehensive quality education is the foundation of health and well-being. In order for people to lead productive and healthy lives, they must possess the necessary knowledge for the prevention of diseases and pathologies, enjoy adequate nutrition, and enjoy good health [7].

Education is a catalyst for development in health-related action. Indeed, the United Nations Educational Scientific and Cultural Organization (UNESCO) 2015 Incheon Declaration develops principles and establishes strategies for the implementation of the Education Goal (SDG4) of the Sustainable Development Goals (SDGs) and confirms that education must develop the skills, values, and attitudes for citizens to enjoy a healthy life, make informed decisions, and address problems at local and global levels [9].

The health emergency caused by COVID-19 has shown that millions of children do not receive necessary vaccines because their parents refuse or because they do not have access to them. It should be remembered that if a child does not receive vaccination properly, not only is his life endangered, but it also affects other children who live with him, their families, and teachers. Consequently, it can be indicated that vaccines are synonymous with education because they improve quality of life and the schooling process. However, regardless of socioeconomic or educational status, there are many people who oppose vaccination in all countries of the world and in all social sectors. Reluctance to vaccinate varies from approbation to absolute refusal, with diverse variations that have been termed “vaccine reluctance” [10].

Currently, in the face of the COVID-19 pandemic, debate has been emerging about the need—or lack thereof—for vaccines, in certain cases, to be mandatory, as well as administrative and judicial procedures to impose mandatory vaccination and cases in which the courts and tribunals can demand it. In some countries, such as Spain, vaccination is voluntary since the law does not explicitly incorporate the duty of vaccination, and therefore, it is not possible to force vaccination. However, there are certain situations that allow the competent public authorities to impose forced vaccination, mainly in the case of epidemics [11].

Likewise, an intense social debate has emerged on the need to introduce regulatory changes that favor the mandatory imposition of vaccination in certain cases, such as highly contagious and serious diseases and when eradication is possible with the adoption of such a coercive measure. This debate has been favored by the public health measures adopted in Italy (where 10 compulsory and 4 highly recommended vaccines have been imposed) and France (where mandatory vaccination of 11 immune preventable diseases has been established). Both decisions are justified by the alarming decline in vaccination rates that had occurred in their respective territories. However, in Spain, as shown by mandatory reports, it is noted that vaccination rates are high [12].

The debate has focused more on health professionals and teachers, as they are high-risk groups, as has been proven in the face of the COVID-19 disease, which is why they form a target group in terms of vaccination, both for reasons of “public health” (protect them from the risk of contracting certain diseases, prevent them from being a source of contagion for third parties, and, at the same time, collaborate on the application of the vaccination schedule) and “occupational health” (protect them from the risk of exposure to contagion or complications of the acquisition of infectious diseases in the workplace and avoid absenteeism). In addition, vaccination is, as for any citizen, also a right for health professionals in all countries [12,13].

If we focus on the educational community, it should be stressed that the majority of teachers and parents do not have easy access to the appropriate information to decide whether it is appropriate to vaccinate children. Therefore, it should be health professionals who inform parents about the benefits and risks of vaccination. Consequently, it should be pediatric teams that collaborate to ensure accurate information, with particular attention given to parents who may have misgivings about vaccines. Indeed, the pediatric team must provide parents with complete information about the vaccines their children may receive, including all licensed and appropriate vaccines and whether or not they are funded by public institutions. Likewise, it is necessary that the pediatric team record in the medical record that parents are informed of all the vaccines recommended for the prevention of diseases. This record or “vaccination card” should be known to teachers so that they have all the information about the vaccination process of the students. In addition, a nurse should be available at all educational centers, and “health education” should be promoted [14,15].

Parents or guardians can refuse vaccination, a problem of maximum topicality we find ourselves facing that is generating an intense debate on the obligatory nature of vaccination. 

Several countries, since November 2021, are adopting forceful legal measures to force vaccination. For example, the Latvian Parliament has approved, as a matter of urgency, a legal change that will allow the dismissal of workers who refuse to be vaccinated against COVID-19. It clearly aims to attempt to halt the uptick in the spread of COVID by allowing employers to suspend workers from employment and subsequently fire them if they are not vaccinated three months after they have received the suspension. In addition, it has approved an amendment stipulating that public sector employees may be laid off if they do not have the COVID certificate or have contracted and recovered from the disease [16].

In November 2021, as announced by health authorities, Costa Rica has become the first country in the world to force children to be vaccinated against COVID-19. The injection will join the long list of basic childhood vaccines that are required by law. In effect, the government of the Central American country has signed an agreement with Pfizer to obtain doses for and address the vaccination of all children under 12 years of age from March 2022. Similarly, in the first week of November, U.S. regulatory bodies approved the Pfizer-BioNTech vaccine for children between the ages of 5 and 11 [17].

In this social context, day by day, the rejection of vaccination has been evidenced in the media and on social networks, generating a growing concern around the world regarding how the problem is being addressed. Consequently, the intensification of vaccine refusal is being assessed as an increasing menace to collective health [18] that can cause immunization campaigns to fail to achieve their desired success [19]. 

Health education has a multidimensional perspective that facilitates precise knowledge, attitudes, and skills, instilling awareness of the determinants of health and enabling training so that it can be carried out with the participation of society as a whole.

The final objective of health education is the transformation of harmful behaviors and the reinforcement of healthy ones, and its fundamental axis is communication, covering aspects related to education, training, research, legislation, policy coordination, and communicative development [20,21]. 

Low educational levels have been associated with greater health problems in the literature [22,23], because a low level of health education can be rooted in various social barriers that hinder access to health services; difficulties in the correct use of medicines; problems of access to adequate health information; or complications in the control of chronic diseases [24].

The research collected in this article was carried out in response to the need to plan actions that favor the vaccination processes of all citizens worldwide as a tool to guarantee individual and collective health. Therefore, it was considered essential to know the levels of awareness of vaccination processes of professionals from essential social sectors (education, health, and economy). The objectives aim to reflect on the measures of mandatory vaccination, to know the importance of teachers being able to demand a regulated vaccination card from students, to study the need for regulation by administrations for compliance with vaccines, and to analyze the importance of health education.

## 2. Materials and Methods

An observational study is proposed through a cross-sectional design of a quantitative nature that aims to study the importance of vaccines in the health of the child population.

### 2.1. Population and Sample

The study sample involves 1000 sharers whose nationalities cover 76 nations. The sample selection was carried out using a non-probabilistic sampling procedure of consecutive type, also known as total enumerative. Professional development in any of the three sectors studied was established as the sole criterion for inclusion—specifically, performing a job within one of the following sectors: health, education, or economy.

**Figure 1:**
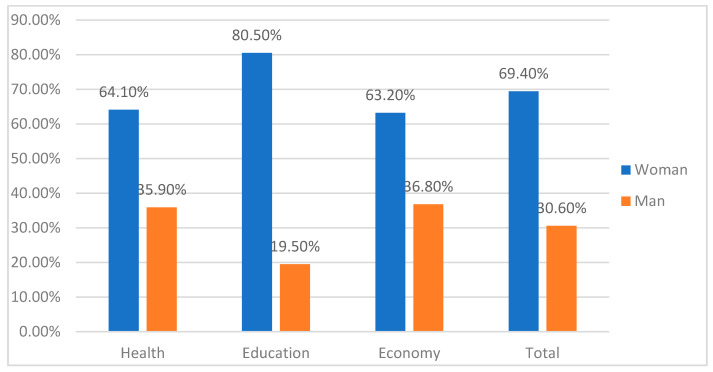
Characteristics of the sample according to sex and sector.

The majority age group in the sample is under 30 years (36.30%), followed by the range between 30 and 44 years (34.80%), while those between 45 and 59 years represent 26.76% of the sample, and those over 60 years represent 2.20% of the total respondents. Figure 1 shows the characteristics of the participants in relation to the sector to which they belong as well as to their sex; of note, they are mostly women.

### 2.2. Instrument

Applying the survey technique, the VACUNASEDUCA questionnaire was used as an instrument [25]. It consists of 12 items with Likert scale distributed into four dimensions, as can be seen in Table 1.

This instrument was developed ad hoc and validated through expert judgment and exploratory factor analysis. Through the first method, the CVI (content validity index) was calculated, whose results for each dimension were D1 = 0.87, D2 = 0.93, and D3 and D4 = 1, with the mean index being 0.96. 

The Kaiser–Meyer–Olkin (KMO) test (0.784) and the Bartlett sphericity test (0.000) showed adequacy for factor analysis. 

The reliability of the instrument used was calculated using Cronbach’s alpha (α), obtaining a mean value of 0.64 for the four dimensions, which is close to the 0.70 set for an acceptable consistency [26].

### 2.3. Variables

Each of the items of the instrument constitutes an ordinal variable, which results in a total of 12 ordinal variables.

A quantitative dependent variable (S3t) was calculated that represents the rank of each of the participants of the 12 items of the instrument. This variable was calculated by adding up the single ordinal punctuations for every participant and dividing by the number of items (12) to typify them.

The study consists of the following variables:

- Sex—dichotomous independent variable with 2 possibilities: male and female.
- Age—polytomous independent variable with 4 possibilities: less than 30, between 30 and 44, between 45 and 59, greater than 60.
- Sector—polytomous independent variable with 3 possibilities: health, education, economy.
- Country—polytomous independent variable with 76 possibilities: Albania, Germany, Andorra, Angola, Saudi Arabia, Algeria, Argentina, Australia, Austria, Bangladesh, Belgium, Bolivia, Bosnia–Herzegovina, Botswana, Brazil, Bulgaria, Cape Verde, Cameroon, Canada, Chile, China, Cyprus, Colombia, Ivory Coast, Cuba, Denmark, Ecuador, United Arab Emirates, Spain, U.S.A., Estonia, Philippines, Finland, France, Gabon, Gambia, Georgia, Greece, Guatemala, Equatorial Guinea, Haiti, Honduras, India, Ireland, Iceland, Israel, Italy, Jamaica, Japan, Jordan, Latvia, Lebanon, Liberia, Luxembourg, Morocco, Mauritius, Mauritania, Mexico, Montenegro, Mozambique, Paraguay, Peru, Poland, Portugal, United Kingdom, Russia, South Africa, Sweden, Switzerland, Thailand, Tanzania, Turkey, Ukraine, Uganda, Venezuela, Zimbabwe.
- Human Development Index (HDI)—polytomous independent variable with 4 possibilities: very high, high, medium, low.
- Continent—polytomous independent variable with 5 possibilities: Europe, America, Asia, Africa, Oceania.

### 2.4. Procedure

The instrument utilized was applied between the months of September 2019 and March 2020. Data collection was carried out by different international institutions such as the WHO office in Geneva (Switzerland); the United Nations (UN); the Pan American Health Organization (PAOH); the Food and Agriculture Organization of the United Nations (FAO); in hospitals, universities, and educational centers in Spain; and in international education and medical congresses.

It was always the same researcher who was in charge of data collection, carried out in a self-administered way and with two versions, Spanish and English. There was no time limitation for its completion, and at all times, the confidentiality of the data collected and the anonymity of all participants were guaranteed. All were of legal age and were adequately informed that their participation was voluntary and that they could abandon the completion of the questionnaire at any time without the need for any justification. The completion of the questionnaire implied implicit acceptance in the study. In addition, in no case was any kind of incentive provided to the participants. 

### 2.5. Data Analysis

Null modeling techniques were used, employing resampling procedures utilizing the Monte Carlo simulation method with the bootstrap method [27], as the sample distribution did not respond to the characteristics of a Gaussian distribution. The term bootstrap is derived from the phrase “to pull oneself up by one’s Bootstrap” from the book *The Surprising Adventures of Baron Munchausen* by Rudolf E. Raspe, and it manifests the very self-sufficiency of the procedure by using only the information available in the data itself, discarding external help from theoretical assumptions or classical models [28]. The bootstrap method belongs to a procedure of resampling of data that performs computer simulation procedures built on the production of a huge number of reiterated samples from the available data and on which the inferential and descriptive statistical evaluation is performed utilizing confidence intervals (CIs) pulled out from the available data.

Reasonably, some authors define these procedures as exhaustive techniques of computer estimation that are built into recent statistical methods, in dispute with the traditional mathematical method [29,30].

In these new techniques, it is possible to dispense with the assumptions related to distributions such as normality, since, instead of assuming a special theoretical distribution, only the original sample is used, and a large number of subsamples are created by simulation that are used to inductively estimate the shape of the sample distribution of the statistics. In this way, it is possible to analyze data derived from unknown distributions so that analytical work is replaced by an empirical procedure of computer-intensive calculation that offers possible solutions when there are no usable formulas to solve the problem by classical statistical procedures [31] (p. 149).

To check for statistically significant dissimilarities, an ANOVA test was performed for independent samples for each independent variable of factor analysis. Through the analysis of the multivariate general linear model, the values of Fisher’s F statistic, the *p*-value level of significance, and the size of the effect assessed by partial eta squared were calculated.

Similar results were obtained in the post hoc tests that were carried out assuming non-equal variances through the Tamhane’s T2, Dunnett’s T3, Games–Howell, and Dunnett’s C statistics, which allowed for the establishment of the direction column in the different ANOVA tables of each of the factors examined.

Likewise, the Spearman and Kendall’s Tau tests yielded very similar results when performing a non-parametric bivariate correlational analysis between the study variables.

## 3. Results

Consistent with the objectives set for the research, the results are detailed. Indeed, in Table 2, high results are shown for dimensions 1 and 2, and item P06, which obtain the higher mean value (M = 2.86, SD = 0.44) (the maximum mean value of the questionnaire as a whole), as well as all outcomes for dimensions 3 and 4 and items P11 and P12, which obtain the lower mean score (M = 1.18, mean SD = 0.42 and 0.43) (the lowest mean value of the instrument as a whole).

In relation to the mean scores for each dimension, the following stand out: dimension 1 (M = 2.81, SD = 0.36), dimension 2 (M = 2.81, SD = 0.31), dimension 3 (M = 1.47, SD = 0.59), and dimension 4 (M = 1.20, SD = 0.41). The mean of the instrument was (M = 2.05, SD = 0.22).

In this paper, we are going to focus more broadly on dimension D3: Regulation and obligation.

Focusing on the results that affect dimension 3, with the independent variables of sex, age, sector, HDI, continent, and country, each variable is highlighted in the following sections.

### 3.1. Analysis of the Incidence of Sex

Table 3 shows the distribution of the sample corresponding to sex (female and male) for dimension 3.

Table 3 shows that the distribution of the sample corresponding to sex is unequal, as the percentage of women (69.4%) is higher than that of men (30.6%).

To examine whether there were dissimilarities corresponding to sex in the instrument, an ANOVA test was carried out for independent samples. The outcomes are reflected in Table 4.

Post-hoc tests show that the mean of men is above that of women in dimension 3, with lower means and therefore with a tendency towards NO, so it could be concluded that women, in general, manifest a lower trend than that of men toward supporting the need for teachers to require students to have a regulated vaccination card and toward the existence of an adequate regulation that supports teachers in the requirement of compliance with vaccination in children.

It should be noted that in item P07, item P08, and dimension 3, statistically significant dissimilarities are shown, although the size of the effect assessed in the ANOVA test by partial eta squared, being less than 0.06, has to be considered as a faint effect [32].

### 3.2. Analysis of the Incidence of Age

Table 5 shows the distribution of the sample by age group as follows: less than 30, between 30 and 44, between 45 and 59, and greater than 60.

In Table 5, it can be seen that the distribution of the sample by age groups is unequal, given that the percentages of the age groups under 30 (36.3%), between 30 and 44 (34.8%), and between 45 and 59 (26.76%) are similar while the percentage of the group greater than 60 (2.2%) is an order of magnitude lower.

In order to determine whether there were dissimilarities corresponding to the age group in the questionnaire, an ANOVA test was carried out for independent samples. The outcomes are shown in Table 6.

Post-hoc tests show that the age group under 30 years of age obtains lower means and has a tendency towards NO. Consequently, it could be deduced that the age group under 30 years of age shows a lower trend toward the need for teachers to require students to have a regulated vaccination card and toward the existence of an adequate regulation that supports teachers in the requirement of compliance with vaccination in children.

Statistically significant dissimilarities are showed in items P07 and P08 and in dimension 3, such that the size of the effect assessed in the ANOVA test by partial eta squared, being greater than 0.06, can be considered as a medium effect [32].

### 3.3. Analysis of the Incidence of Sector

Table 7 shows the distribution of the sample by sector, divided into health, education, and economy.

The distribution of the sample by sector is uneven, as can be seen in Table 7. Indeed, the percentage of the health sector (55.4%) constitutes more than one-half of the sample, whereas the percentage of the education sector (32.9%) constitutes one-third of the sample, and the percentage of the economy sector (11.7%) makes up a smaller part of the sample.

To determine whether there were dissimilarities corresponding to the sector in the instrument, an ANOVA test was carried out for independent samples. The outcomes are shown in Table 8.

Post-hoc tests show that the mean of the education sector is above the health sector in dimension 3, with higher means and consequently a tendency towards YES. Therefore, it could be concluded that the education sector, in general, has a higher tendency than the health sector toward considering the need for teachers to require students to have a regulated vaccination card and the existence of an adequate regulation that supports teachers in the requirement of compliance with vaccination in children. The means of the economy sector are in the middle, showing significant dissimilarities with the education sector and insignificant ones with the health sector.

Statistically significant dissimilarities are shown in items P07 and P08 and in dimension 3, although the effect size assessed in the ANOVA test by partial eta squared, being less than 0.06, has to be considered as faint in items P07 and P08, while in dimension 3, it being greater than 0.06, it can be considered to have a medium effect size [32].

### 3.4. Analysis of the Incidence by Human Development Index (HDI)

Table 9 shows the distribution of the sample by Human Development Index (HDI), with the categories of very high, high, medium, and low.

The distribution of the sample by Human Development Index (HDI) is unequal. In fact, the percentage of group I1 = Very high (87.3%) is an order of magnitude higher than the percentage of groups I2 = High (8.5%), I3 = Medium (3.1%), and I4 = Low (1.1%), which are of a similar order of magnitude. Reasonably, in order to determine whether there were dissimilarities corresponding to the Human Development Index (HDI) in the instrument, an ANOVA test was carried out for independent samples. The outcomes are shown in Table 10.

Post-hoc tests show that, in dimension 3, the mean of the rest of the groups is above that of the very high Human Development Index (HDI) group, and with a tendency towards NO, it could be concluded that the very high Human Development Index (HDI) group offers a lower trend than the rest of the groups toward the need for teachers to demand from students a regulated vaccination card and toward the existence of an adequate regulation that supports teachers in the requirement of compliance with vaccination in children. 

Statistically significant dissimilarities are showed in items P07 and P08 and in dimension 3, even though the effect size assessed in the ANOVA test by partial eta squared, being less than 0.06, has to be considered as a faint effect [32].

### 3.5. Analysis of the Incidence by Continent

Table 11 shows the distribution of the sample by continent: Europe, America, Asia, Africa, and Oceania. 

Table 11 shows that the distribution of the sample by continent is unequal; the percentage Europe (83%) is an order of magnitude higher than the percentage of the rest of the continents of America (9.3%), Asia (4%), Africa (3.5%), and Oceania (2%), which are of a similar order of magnitude.

In order to determine whether there were dissimilarities corresponding to continent, an ANOVA test was carried out for independent samples, eliminating Oceania from the study since only two participants from that continent concluded the survey, which makes it inadequate for statistical study utilizing resampling techniques through the Monte Carlo simulation method with the bootstrap algorithm. The outcomes are shown in Table 12.

It can be observed that the post-hoc tests show that the mean of the respondents from the rest of the continents is above that of the continent of Europe in dimension 3, with lower means and with a clear tendency towards NO, so it could be concluded that the respondents of the continent of Europe manifest a lower trend than the respondents of the rest of the continents toward the need for teachers to require students to provide a regulated vaccination card and toward the existence of an adequate regulation that supports teachers in the requirement of compliance with vaccination in children.

Statistically significant dissimilarities are showed in items P07 and P08 and in dimension 3; the effect size assessed in the ANOVA test by partial eta squared, being greater than 0.06, can be considered as a medium effect [32].

### 3.6. Analysis of the Incidence by Country

Table 13 shows the number of respondents per country in the sample.

In order to facilitate the inferential statistical analysis, the seven countries with the most respondents were Germany, Spain, France, Italy, Portugal, the United Kingdom, and the U.S.A., which constitute 75.7% of the total sample. The distribution of the sample for these seven countries is collected in Table 14.

As can be seen in Table 14, the distribution of the sample by country is unequal; the percentage of E = Spain (59.6%) is an order of magnitude higher than the percentage of the rest of the countries analyzed, namely Germany (2.8%), France (4.9%), Italy (2.7%), Portugal (1.9%), the United Kingdom (5.9%), and the U.S.A. (1.7%), which are of a similar order of magnitude.

To determine whether there were dissimilarities corresponding to the country studied, an ANOVA test was carried out for independent samples. The outcomes are reflected in Table 15.

Post-hoc tests show that the mean of the rest of the countries is above that of the country of Spain in dimension 3, with lower means. Consequently, it could be concluded that the country of Spain shows a lower trend than the respondents of the rest of the countries toward the need for teachers to require students to have a regulated vaccination card and toward the existence of an adequate regulation that supports teachers in the requirement of compliance with vaccination in children 

Statistically significant dissimilarities are shown in items P07 and P08 and in dimension 3, even though the size of the effect assessed in the ANOVA test by partial eta squared, being greater than 0.06, can be considered as a medium effect in items P07 and P08, while in dimension 3, with its partial eta squared being greater than 0.14, it can be considerate as a large effect [32].

### 3.7. Correlational Analysis

Table 16 shows spearman’s matrix of non-parametric bivariate correlations, in which it is evident that the country variable does not correlate significantly with any of the other variables of the study, while we observed a significant positive correlation between the variables sex, age, HDI, and continent and a significant negative correlation of the variable sector with the other variables of the study. All correlations, when presenting a correlation coefficient between 0.10 and 0.30, can be considered to have a faint effect size, except the correlation between the HDI variables and continent, which, by presenting a correlation coefficient greater than 0.50, can be considered to have a heavy effect size [32].

## 4. Discussion

The gaps in the legislation of the various countries of the obligatory nature of vaccines at different ages have been highlighted. This situation is aggravated by the expansion of infections caused by COVID-19 and its variants. For this reason, some European countries are assessing the need for mandatory vaccination for health personnel, teachers, students, staff of nursing homes, etc. Indeed, the debate on mandatory vaccination is very much alive around the world. In addition, currently, mandatory vaccination is provoking significant protests in European countries such as Greece, France, Germany, etc., claiming that fundamental rights could be violated. 

In the field of health, children and adolescents have not suffered the consequences of COVID-19 with the same intensity as adults; however, they are a vulnerable group because their routines and habits of life have been altered due to the measures adopted by the rulers of their countries. Indeed, the consequences of confinement in childhood impact individual aspects, such as food and physical and mental health, and social aspects, such as education, coexistence between equals, and leisure. In addition, children have been exposed to conditions of adversity, such as violence, abuse, etc. Likewise, they have suffered a bombardment of information, not always correct, from their family, and the media and social networks [33,34,35,36,37,38,39,40].

In several studies, it has been proven that the most affected have been the most vulnerable groups socially and personally, proving an increase in anxiety and intense emotions, disturbing their social and emotional well-being. Especially affected have been children who possess attention deficit hyperactivity disorders [41,42,43], children with autism spectrum disorder [44], and “gifted” children [40,45,46,47,48].

It can be indicated that all mandatory vaccinations are financed by the health system of the concerned country. In addition, the situation currently differs little from that of 2010, when 15 European countries included some mandatory vaccine in their calendars, the most frequently involved being those for diphtheria, tetanus, poliomyelitis, and hepatitis B, followed by those of measles, rubella, mumps, whooping cough, Haemophiles influenzae, and tuberculosis. Likewise, each country has approached the phenomenon of the fall in vaccination coverage and the appearance of outbreaks of vaccine-preventable diseases differently. 

The consequences generated in the student body have further affected vulnerable groups due to the lack of equity in access to educational and social services. Consequently, international action organizations such as the World Health Organization (WHO), United Nations International Children’s Emergency Fund (UNICEF), UNESCO, OECD, the Council of Europe, and the European Commission (EC) have stressed the importance of schools incorporating health education into their curricula as the main tool to develop healthy lifestyle habits, increase the quality of life of schoolchildren, and consequently work on building a more inclusive and healthier world [49,50].

It should be stressed that mandatory vaccination has always been linked to controversy, and there are no short-term changes in sight. For the regulatory route by itself without other accompanying measures, it is a path of little scope in the medium–long term since a sustained improvement in compliance with vaccination programs is not always achieved. In addition, its implementation is complicated, polarizing points of view in the social debate, and can lead to less trust in authorities and health professionals. However, in a timely manner, it could be an acceptable tactical option to prevent high-risk situations when there are no other feasible alternatives in the short term.

Parents or guardians can refuse vaccination, a problem of maximum topicality that we find ourselves facing. In these cases, in which there is a refusal of parents or guardians to vaccinate their children or represented wards; before any legal consideration, information measures should be developed on the risks and benefits of vaccination, as well as on the risks of non-vaccination. Reasonably, the doctor–patient relationship should always be kept open to facilitate, where appropriate, the reconsideration of the rejection of vaccines and their subsequent acceptance. Likewise, it is necessary that the refusal of parents or guardians to vaccinate their children or represented wards is reflected, at least, in the clinical history, with an indication that this refusal is maintained despite having been informed of both the risks/benefits of vaccination and the risks of non-vaccination. This registry could be used in the event that it is necessary to prove whether there was sufficient information about the possible risks of the vaccine [2].

Debate on the consequences of parents refusing to vaccinate their school-age children, a conflict between two fundamental rights, has arisen. On the one hand, there is the right to ideological freedom, privacy, and physical integrity of parents and minors who do not opt for vaccination, and on the other, the right to health of the rest of the children who attend the same school.

Rejection of vaccination is a problem that affects all global citizens and demands awareness, because having an effective vaccine is not an individual issue, but rather one in which the more citizens are vaccinated, the more a so-called “herd immunity” will be achieved and the virus will stop circulating [51].

We agree with other research [52,53,54] on the importance of governments making an effort so that citizens know and understand the consequences of reluctance to vaccinate, its determining factors, and the challenges it poses.

## 5. Conclusions

In the research, it has been shown that women and those under 30 years of age are the least likely participants from whom teachers can demand a regulated vaccination card, and regarding the need for adequate regulation by administrations of the precise actions for compliance with vaccines, the health sector is the greatest defender of its demand, even above the education sector and the economy sector. Likewise, participants from the rest of the groups are above the very high HDI group. In addition, Europe is the continent that has the lowest means, with Spain being where participants express a lower trend than respondents from the rest of the countries.

Some sectors of society defend the mandatory vaccination of teachers and, in some cases, of students. However, research has shown that younger people with very high HDI and European level are those who least value the need for teachers to require a regulated vaccination card and for administrations to adequately regulate compliance with vaccination in schools. This is entirely understandable, as it is the group that benefits most from the vaccination processes and has the most information. All of this leads us to conclude that there is a need for adequate information campaigns on the importance of vaccination and that all children in the world can enjoy its benefits without discrimination of any kind.

The current global pandemic situation has justified the need for greater investment in the field of education and in the biomedical area. The consequences of the SARS-CoV-2 pandemic are closely related to the level of investment and development (R&D), so one of the factors that is causing the slow progress of the epidemic response is the small number of vaccinated populations worldwide and its unequal form of distribution in the various countries.

We believe that the purpose of health education in schools, along the lines proposed by the Pan American Health Organization [55], focuses on the training of students from the earliest age in the knowledge, attitudes, and skills that contribute to the acquisition of healthy lifestyle habits, considering physical, psychic, and social needs. In addition, these skills must be acquired in an integral way, promoting their adaptation to a complex and changing society.

It is necessary to stress the importance of promoting public health policies that implement health education programs, with special attention given to vaccination processes in the general population.

On the basis of the above, it would be necessary to call on the competent authorities to clearly regulate the legal framework affecting vaccination in order to guarantee the rights of all citizens and, especially, the rights of minors, particularly in the most vulnerable groups and countries.

In practice, the most serious risks of non-vaccination are often not reported, and written consent is not required. Therefore, it would be beneficial to know that consent and the vaccination card are very important evidence accurately reflecting the reality of the information so that the legal risk could be reduced considerably if the information instruments are improved and there is evidence of it in the medical record.

The administrations of the countries of the world should prepare to deal with mandatory vaccination and its regulation and, in addition, reduce risk factors. This task is not easy because it is not just any incident or crisis, but an event that goes beyond institutional and individual assets and strategies.

In summary, the most significant conclusion of the work carried out shows that health education is one of the main tools for societies and citizens to access universal rights. In addition, health promotion implies the improvement of the health skills of the subjects, as well as the environment and society in which they live. The results of the research have served to make concrete curricular proposals on health education in teacher training centers.

Among the limitations of the study, it is necessary to refer to the fact that the elements of the sample did not meet the conditions to be considered a sample with a normal distribution, and that is why we used the bootstrap technique. On the other hand, the type of consecutive sampling used decreases the external validity of the study. 

It is necessary to indicate that the data collection coincided with the previous and initial moments of the COVID-19 pandemic and therefore was underway before the existence of vaccines against this virus. However, what could be considered as a limitation has in practice strengthened the research and ratified the conclusions drawn. In this sense, it should also be noted that this pandemic situation and the evolution of events related to vaccination processes could have influenced the modification of the opinions and perceptions that the participants had about the items of the applied questionnaire.

Finally, the selection of topics could be considered a limitation. In addition, due to the novel nature of the study, it is necessary to continue research deepening the importance of the training and social commitment of parents so that they become active engines in favor of vaccination processes in their communities.

## Figures and Tables

**Table 1:** Structure of the instrument in dimensions.

Dimensions	Items
D1 = Awareness and regulation	1–2
D2 = Education and teachers	3–4 to 5–6
D3 = Regulation and obligation	7–8
D4 = Consequences and risks	9–10–11–12

**Table 2:** Count after application of the questionnaire.

		Scale (*n*)					95% CI			95% CI	
Dimension (D)	Item	1	2	3	*n*	M	Lower	Upper	SD	Lower	Upper
	P01	26	174	800	1000	2.77	2.75	2.80	0.48	0.44	0.51
	P02	14	132	854	1000	2.84	2.81	2.86	0.40	0.37	0.44
D1 = Awareness and regulation	D1t					2.81	2.78	2.83	0.36	0.34	0.38
	P03	34	131	835	1000	2.80	2.77	2.83	0.48	0.44	0.52
	P04	27	152	821	1000	2.79	2.77	2.82	0.47	0.43	0.50
	P05	35	139	826	1000	2.79	2.76	2.82	0.49	0.44	0.52
	P06	35	74	891	1000	2.86	2.83	2.88	0.44	0.40	0.49
D2 = Education and teachers	D2t					2.81	2.79	2.83	0.31	0.28	0.33
	P07	616	183	201	1000	1.59	1.53	1.64	0.80	0.78	0.83
	P08	701	240	59	1000	1.36	1.32	1.40	0.59	0.56	0.62
D3 = Regulation and obligation	D3t					1.47	1.44	1.51	0.59	0.56	0.61
	P09	791	174	35	1000	1.24	1.21	1.28	0.50	0.47	0.54
	P10	828	149	23	1000	1.20	1.17	1.22	0.45	0.41	0.48
	P11	840	143	17	1000	1.18	1.15	1.20	0.42	0.39	0.46
	P12	836	147	17	1000	1.18	1.16	1.21	0.43	0.39	0.46
D4 = Consequences and risks	D4t					1.20	1.18	1.23	0.41	0.37	0.44
Total	S3t					2.05	2.04	2.06	0.22	0.20	0.23

**Table 3:** Sex count of the participating sample for dimension D3.

		SEX						
		Man *n* = 306 = 30.6%			Woman *n* = 694 = 69.4%			
Dimension (D)	Item	1	2	3	1	2	3	*n*
D3 = Regulation and obligation	P07	157	60	89	459	123	112	1000
	P08	193	87	26	508	153	33	1000

**Table 4:** ANOVA for independent samples based on sex for dimension D3.

		Man			95% CI		Woman		95% CI				
Dimension (D)	Item	M	Lower-Upper	SD	Lower-Upper	M	Lower-Upper	SD	Lower-Upper	F	*p*	Eta2	Direction
	P07	1.78	1.68–1.88	0.87	0.83–0.90	1.50	1.44–1.56	0.76	0.72–0.79	26.02	<0.01	0.03	W < M
	P08	1.45	1.39–1.53	0.65	0.59–0.70	1.32	1.28–1.36	0.56	0.52–0.60	11.86	<0.01	0.01	W < M
D3 = Regulation and obligation	D3t	1.62	1.55–1.69	0.64	0.60–0.68	1.41	1.37–1.45	0.55	0.52–0.58	27.17	<0.01	0.03	W < M

**Table 5:** Count by age group of the participating sample for dimension D3.

		AGE												
		Less than 30 *n* = 363 = 36.3%			Between 30 and 44 *n* = 348 = 34.8%			Between 45 and 59 *n* = 267 = 26.76%			Greater than 60 *n* = 22 = 2.2%			
Dimension (D)		1	2	3	1	2	3	1	2	3	1	2	3	*n*
D3 = Regulation and obligation	P07	297	26	40	188	83	77	120	71	76	11	3	8	1000
	P08	315	36	12	232	94	22	141	102	24	13	8	1	1000

**Table 6:** ANOVA for independent samples by age group for dimension D3.

Item	M	E1 = <30 L-U	SD	95% CI	M	E2 = 30–44 L-U	SD	95% CI	*p*	Eta2	Direction
P07	1.29	1.22–1.36	0.65	0.58–0.72	1.68	1.60–1.77	0.81	0.77–0.85	0.00	0.08	E1 < E2.E3
P08	1.17	1.12–1.21	0.45	0.38–0.52	1.40	1.33–1.46	0.61	0.55–0.65	0.00	0.07	E1 < E2 < E3
D3t	1.23	1.18–1.28	0.46	0.40–0.51	1.54	1.48–1.60	0.59	0.55–0.62	0.00	0.11	E1 < E2 < E3.E4
Item	M	E3 = 45–59 L-U	SD	95% CI	M	E4 = >60 L-U	SD	95% CI	*p*	Eta2	Direction
P07	1.84	1.73–1.93	0.84	0.80–0.87	1.86	1.45–2.29	0.94	0.76–1.00	0.00	0.08	E1 < E2.E3
P08	1.56	1.48–1.64	0.65	0.60–0.70	1.45	1.22–1.73	0.60	0.43–0.75	0.00	0.07	E1 < E2 < E3
D3t	1.70	1.62–1.78	0.63	0.59–0.67	1.66	1.39–1.96	0.64	0.48–0.77	0.00	0.11	E1 < E2 < E3.E4

Note: L = lower; U = upper.

**Table 7:** Count by sector of the participating sample for dimension D3.

		Sector									
		Health *n* = 554 = 55.4%			Education *n* = 329 = 32.9%			Economy *n* = 117 = 11.7%			
Dimension (D)	Item	1	2	3	1	2	3	1	2	3	*n*
D3 = Regulation and obligation	P07	302	123	129	255	32	42	59	28	30	1000
	P08	356	159	39	279	44	6	66	37	14	1000

**Table 8:** ANOVA for independent samples by sector for dimension D3.

Item	M	Health L-U	SD	95% CI L-U	*p*	Eta2	Direction
P07	1.69	1.62–1.76	0.83	0.79–0.85	0.00	0.04	S2 > S1.S3
P08	1.43	1.38–1.48	0.62	0.58–0.66	0.00	0.05	S2 > S1.S3
D3t	1.56	1.51–1.61	0.60	0.57–0.63	0.00	0.06	S2 > S1.S3
Item	M	Education L-U	SD	95% CI L-U	*p*	Eta2	Direction
P03	1.35	1.28–1.43	0.70	0.62–0.76	0.00	0.04	S2 > S1.S3
P04	1.17	1.13–1.22	0.42	0.36–0.48	0.00	0.05	S2 > S1.S3
D3t	1.26	1.21–1.31	0.47	0.42–0.52	0.00	0.06	S2 > S1.S3
Item	M	Economy L-U	SD	95% CI L-U	*p*	Eta2	Direction
P03	1.75	1.60–1.91	0.84	0.77–0.89	0.00	0.04	S2 > S1.S3
P04	1.56	1.43–1.68	0.70	0.61–0.77	0.00	0.05	S2 > S1.S3
D3t	1.65	1.53–1.77	0.66	0.59–0.72	0.00	0.06	S2 > S1.S3

Note: L = lower; U = upper.

**Table 9:** Human Development Index (HDI) count for dimension D3.

		HDI												
		Very high *n* = 873 = 87.3%			High *n* = 85 = 8.5%			Medium *n* = 31 = 3.1%			Low *n* = 11 = 1.1%			
Dimension (D)		1	2	3	1	2	3	1	2	3	1	2	3	*n*
D3 = Regulation and obligation	P07	564	157	152	36	18	31	14	4	13	2	4	5	1000
	P08	638	190	45	37	38	10	21	6	4	5	6	0	1000

**Table 10:** ANOVA for independent samples by Human Development Index (HDI) for dimension D3.

Item	M	Very high L-U	SD	95% CI L-U	M	High L-U	SD	95% CI L-U	*p*	Eta2	Direction
P07	1.53	1.48–1.58	0.77	0.74–0.80	1.94	1.74–2.13	0.89	0.83–0.94	0.00	0.04	I1 < I2 < I3 < I4
P08	1.32	1.28–1.36	0.57	0.53–0.61	1.68	1.54–1.83	0.68	0.58–0.75	0.00	0.03	I1 < I3 < I4 < I2
D3t	1.42	1.39–1.47	0.57	0.54–0.60	1.81	1.68–1.94	0.60	0.53–0.66	0.00	0.05	I1 < I3 < I2 < I4
Item	M	Medium L-U	SD	95% CI L-U	M	Low L-U	SD	95% CI L-U	*p*	Eta2	Direction
P07	1.97	1.65–2.29	0.95	0.84–1.00	2.27	1.80–2.73	0.79	0.45–0.98	0.00	0.04	I1 < I2 < I3 < I4
P08	1.45	1.22–1.70	0.72	0.51–0.87	1.55	1.25–1.86	0.52	0.38–0.55	0.00	0.03	I1 < I3 < I4 < I2
D3t	1.71	1.50–1.92	0.62	0.48–0.72	1.91	1.55–2.25	0.58	0.32–0.71	0.00	0.05	I1 < I3 < I2 < I4

Note: L = lower; U = upper.

**Table 11:** Count by continent of the participating sample for dimension D3.

		Continent															
		Europe *n* = 830 = 83%			America *n* = 93 = 9.3%			Asia *n* = 40 = 4%			Africa *n* = 35 = 3.5%			Oceania *n* = 2 = 2%			
Dimension (D)		1	2	3	1	2	3	1	2	3	1	2	3	1	2	3	*n*
D3 = Regulation and obligation	P07	558	146	126	29	24	40	14	5	21	14	8	13	1	0	1	1000
	P08	624	169	37	38	43	12	18	14	8	20	14	1	1	0	1	1000

**Table 12:** ANOVA for independent samples by continent for dimension D3.

Item	M	Europe L-U	SD	95% CI L-U	M	America L-U	SD	95% CI L-U	*p*	Eta2	Direction
P07	1.48	1.43–1.53	0.74	0.71–0.78	2.12	1.94–2.29	0.86	0.79–0.91	0.00	0.09	C1 < C2.C3.C4
P08	1.29	1.26–1.33	0.54	0.51–0.58	1.72	1.59–1.86	0.68	0.60–0.75	0.00	0.07	C1 < C2.C3
D3t	1.39	1.35–1.42	0.54	0.51–0.57	1.92	1.80–2.05	0.64	0.56–0.69	0.00	0.11	C1 < C2.C3.C4
Item	M	Asia L-U	SD	95% CI L-U	M	Africa L-U	SD	95% CI L-U	*p*	Eta2	Direction
P07	2.18	1.87–2.45	0.93	0.83–0.98	1.97	1.68–2.29	0.89	0.78–0.96	0.00	0.09	C1 < C2.C3.C4
P08	1.75	1.51–2.00	0.78	0.63–0.87	1.46	1.27–1.66	0.56	0.46–0.66	0.00	0.07	C1 < C2.C3
D3t	1.96	1.74–2.15	0.64	0.52–0.74	1.71	1.51–1.92	0.60	0.51–0.66	0.00	0.11	C1 < C2.C3.C4

Note: L = lower; U = upper.

**Table 13:** Number of respondents per country of the participating sample.

Number of Respondents by Country							
Country	*n*	Country	*n*	Country	*n*	Country	*n*
Albania	3	Chile	4	Guatemala	3	Mexico	10
Germany	28	China	6	Guinea Equatorial	4	Montenegro	2
Andorra	2	Cyprus	4	Haiti	1	Mozambique	1
Angola	5	Colombia	13	Honduras	2	Paraguay	2
Arabia Saudi	4	Ivory Coast	2	India	6	Peru	2
Argelia	2	Cuba	3	Ireland	9	Poland	1
Argentina	6	Denmark	8	Islandic	2	Portugal	19
Australia	2	Ecuador	6	Israel	3	United Kingdom	21
Austria	6	United Arab Emirates	1	Italy	27	Russia	2
Bangladesh	2	Spain	596	Jamaica	3	South Africa	3
Belgium	6	USA	17	Japan	4	Sweden	2
Bolivia	5	Estonia	1	Jordanian	2	Switzerland	11
Bosnia- Herzegovina	1	Philippines	5	Leetonia	1	Thailand	3
Botswana	1	Finland	4	Lebanon	3	Tanzania	1
Brazil	8	France	49	Liberia	2	Turkey	2
Bulgaria	6	Gabon	1	Luxemburg	6	Ukraine	1
Cape Verde	1	Gambia	1	Morocco	1	Uganda	2
Cameroon	4	Georgia	2	Mauritius	1	Venezuela	2
Canada	6	Greece	8	Mauritania	1	Zimbabwe	3

**Table 14:** Country count of the participating sample for dimension D3.

Dimension (D)	Item	Country									*n*
D3 = Regulation and obligation		Germany *n* = 28 = 2.8%			Spain *n* = 596 = 59.6%			France *n* = 49 = 4.9%			
		1	2	3	1	2	3	1	2	3	
	P07	12	9	7	454	78	64	20	17	12	673
	P08	14	11	3	502	87	7	29	16	4	673
		Italy *n* = 27 = 2.7%			Portugal *n* = 19 = 1.9%			United Kingdom *n* = 59 = 5.9%			
		1	2	3	1	2	3	1	2	3	
	P07	12	11	4	11	4	4	12	6	3	67
	P08	15	10	2	11	6	2	15	6	0	67
		U.S.A. *n* = 17 = 1.7%									
		1	2	3							
	P07	2	3	12							17
	P08	5	6	6							17
							P07	TOTAL			757
							P08	TOTAL			757

**Table 15:** ANOVA for independent samples by country (M, SD from Germany, Spain, France, Italy, Portugal, United Kingdom, and U.S.A.) for dimension D3.

Item	M	Germany L-U	SD	95% CI L-U	M	Spain L-U	SD	95% CI L-U	*p*	Eta2	Direction
P07	1.821	1.50–2.15	0.82	0.64–0.92	1.346	1.30–1.40	0.66	0.61–0.71	0.00	0.11	E < R < P < I < A.F < U
P08	1.607	1.36–1.88	0.69	0.50–0.81	1.169	1.14–1.20	0.41	0.36–0.45	0.00	0.13	E < R < F < I.P < A < U
D3t	1.714	1.45–1.98	0.70	0.56–0.79	1.258	1.22–1.29	0.44	0.40–0.47	0.00	0.16	E < R < P < I < F < A < U
Item	M	France L-U	SD	95% CI L-U	M	Italy L-U	SD	95% CI L-U	*p*	Eta2	Direction
P07	1.837	1.62–2.08	0.80	0.70–0.88	1.704	1.43–1.97	0.72	0.56–0.85	0.00	0.11	E < R < P < I < A.F < U
P08	1.490	1.32–1.70	0.65	0.51–0.77	1.519	1.27–1.77	0.64	0.46–0.78	0.00	0.13	E < R < F < I.P < A < U
D3t	1.663	1.50–1.84	0.58	0.48–0.67	1.611	1.40–1.84	0.56	0.40–0.68	0.00	0.16	E < R < P < I < F < A < U
Item	M	Portugal L-U	SD	95% CI L-U	M	United Kingdom L-U	SD	95% CI L-U	*p*	Eta2	Direction
P07	1.632	1.27–2.00	0.83	0.52–0.97	1.571	1.25–1.94	0.75	0.47–0.91	0.00	0.11	E < R < P < I < A.F < U
P08	1.526	1.24–1.83	0.70	0.45–0.86	1.286	1.10–1.50	0.46	0.31–0.51	0.00	0.13	E < R < F < I.P < A < U
D3t	1.579	1.29–1.89	0.67	0.45–0.80	1.429	1.22–1.66	0.51	0.31–0.62	0.00	0.16	E < R < P < I < F < A < U
Item	M	USA L-U	SD	95% CI L-U					*p*	Eta2	Direction
P07	2.588	2.22–2.92	0.71	0.29–0.93					0.00	0.11	E < R < P < I < A.F < U
P08	2.059	1.64–2.46	0.83	0.62–0.94					0.00	0.13	E < R < F < I.P < A < U
D3t	2.324	1.97–2.65	0.71	0.43–0.86					0.00	0.16	E < R < P < I < F < A < U

Note: L = lower; U = upper.

**Table 16:** Spearman’s matrix of non-parametric bivariate correlations.

		Age	Sex	HDI	Continent	Sector	Country
Age	Correlation Coefficient	1.000	0.234 **	0.206 **	0.256 **	−0.209 **	0.014
	Sig. (2-tailed)		<0.001	<0.001	<0.001	<0.001	0.666
Sex	Correlation Coefficient	0.234 **	1.000	0.120 **	0.127 **	−0.096 **	−0.005
	Sig. (2-tailed)	<0.001		<0.001	<0.001	0.002	0.879
HDI	Correlation Coefficient	0.206 **	0.120 **	1.000	0.813 **	−0.193 **	0.011
	Sig. (2-tailed)	<0.001	<0.001		<0.001	<0.001	0.725
Continent	Correlation Coefficient	0.256 **	0.127 **	0.813 **	1.000	−0.211 **	<0.001
	Sig. (2-tailed)	<0.001	<0.001	<0.001		<0.001	1.000
Sector	Correlation Coefficient	−0.209 **	−0.096 **	−0.193 **	−0.211 **	1.000	−0.042
	Sig. (2-tailed)	<0.001	0.002	<0.001	<0.001		0.189
Country	Correlation Coefficient	0.014	−0.005	0.011	<0.001	−0.042	1.000
	Sig. (2-tailed)	0.666	0.879	0.725	1.000	0.189	

** Correlation is significant at the 0.01 level (2-tailed).

## Data Availability

Due to the anonymity and confidentiality of the data obtained, the authors have not reported any of the data obtained, the purpose of which is exclusively the development of this research.
